# Beyond Excision: Cryotherapy as a Non-surgical Treatment for Palatal Solitary Neurofibroma

**DOI:** 10.7759/cureus.57699

**Published:** 2024-04-06

**Authors:** Jyh-Kwei J Chen

**Affiliations:** 1 Department of Dentistry, Division of Oral and Maxillofacial Surgery, Chang Gung Memorial Hospital, Taipei, TWN; 2 College of Medicine, Chang Gung University, Taoyuan, TWN

**Keywords:** freeze-thawing, neurofibromatosis type 1 (nf-1), liquid nitrogen spray, oral cryotherapy, solitary neurofibroma

## Abstract

Palatal solitary neurofibromas (SNFs), not linked to neurofibromatosis type 1, are uncommon. A 45-year-old female with a palatal SNF underwent non-surgical treatment using liquid nitrogen cryotherapy (LNC). The lesion, initially 9 x 8 x 3 mm, was treated with two 1-2 minute freeze-thaw cycles, progressively extended to two 2-2 minute freeze-thaw cycles to address the refractoriness. After four LNC sessions, the lesion resolved without recurrence at five months. This case demonstrates LNC’s efficacy as a surgical alternative for palatal SNF, offering a non-invasive option for patients declining surgery. The positive outcome warrants further research into LNC’s role in managing similar benign lesions.

## Introduction

Neurofibromas are benign tumors that arise from the peripheral nerve sheath, typically composed of Schwann cells, perineural-like cells, and fibroblasts. They can present as solitary lesions or as part of genetic conditions such as neurofibromatosis types 1 (NF1) and 2 (NF2). Solitary neurofibromas in the oral cavity, including those on the palate, are rare and usually not associated with NF1 or NF2. The classification includes dermal neurofibroma, which arises from a single peripheral nerve and is generally sporadic; plexiform neurofibroma, which involves multiple nerve bundles and is associated with NF1, potentially leading to malignancy; and NF2-associated tumors, which are characterized by bilateral vestibular schwannomas, meningiomas, ependymomas, and other nerve sheath tumors [1].

Oral solitary neurofibromas (SNFs) are relatively uncommon, accounting for approximately 0.046% of all oral lesions. They typically present as non-tender, submucosal masses [2]. The tongue is the most common site, accounting for about 26% of head and neck neurofibromas. The buccal mucosa is another frequent location. The palate, including both the hard and soft palate, represents approximately 8% of cases. Less commonly, oral SNFs are found in the floor of the mouth, gingiva, and alveolar ridge, which together make up about 2% of cases. When an oral SNF is detected, it is crucial to conduct an evaluation to rule out NF1, which may also present with oral symptoms [1]. This wide range of oral distribution highlights the importance of a thorough examination for proper diagnosis and management [3].

In the context of neurofibromas, surgical excision remains the gold standard due to its proven effectiveness and low recurrence rate. However, for patients who are averse to surgery, cryotherapy has shown success in treating tracheal neurofibromas [4]. Additionally, the oral selective MEK inhibitor, Selumetinib, disrupts signals promoting uncontrolled cell growth by targeting the overactive RAS pathway in patients with NF1-related plexiform neurofibromas [5]. Treatment options for oral SNFs include conventional excision surgery and laser excision [6]. Indications for oral SNF removal include discomfort, diagnostic uncertainty, or cosmetic concern.

To address the patient's reluctance to undergo SNF excision surgery, cryotherapy is used as an alternative therapeutic option. Cryotherapy, known for its cost-effectiveness and non-invasive nature, has long been employed in various medical disciplines [7-10]. The efficacy of cryotherapy for palatal SNF remains unexplored. Here, we present a 45-year-old female with palatal SNF who opted for non-surgical treatment using liquid nitrogen cryotherapy (LNC).

## Case presentation

**Figure 1:**
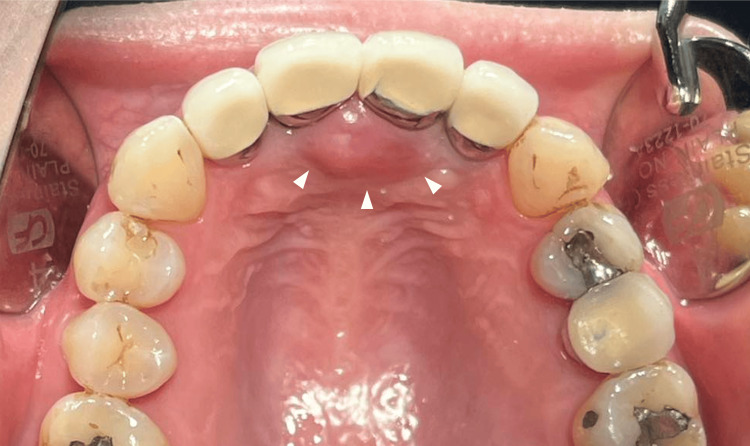
The palatal solitary neurofibroma over incisive papilla An ovoid palatal lesion measuring 9 × 8 × 3 mm, characterized by a smooth surface and dark pink coloration (white arrow heads).

In February 2023, a 45-year-old woman presented with a palatal tumor, noting a three-year swelling history. Upon oral examination, a non-tender lesion with a smooth surface was identified. It exhibited a dark pink hue and an ovoid shape, measuring 9 x 8 x 3 mm. The lesion was soft to touch and located over the incisive papilla (Figure 1).

**Figure 2:**
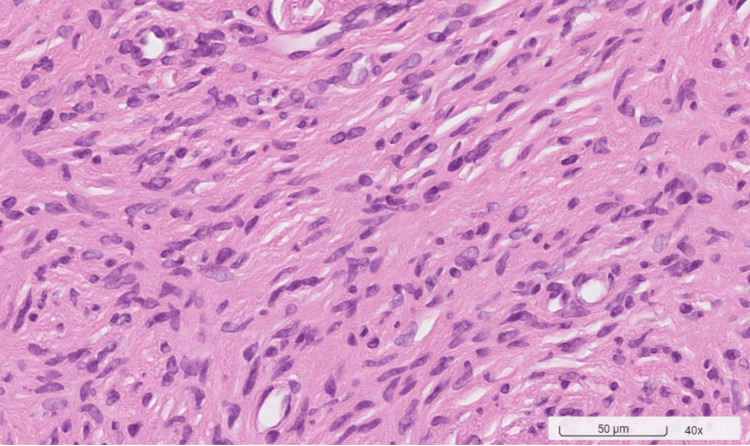
Histopathology revealing a spindle cell tumor of unknown origin (hematoxylin and eosin stain, original magnification x40) The histopathological examination reveals the tumor’s spindle cellular composition without a definitive cellular arrangement pattern.

**Figure 3:**
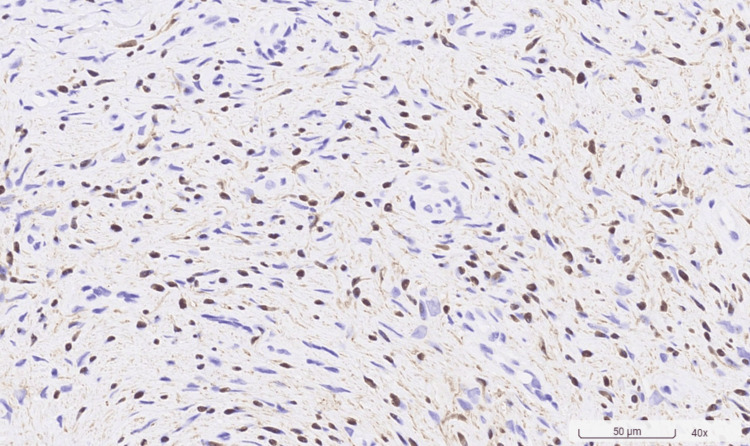
Immunohistochemistry staining showing focal positivity for S100 (polyclonal) (original magnification x40) Focal positivity for S100 protein indicates Schwann cell involvement.

**Figure 4:**
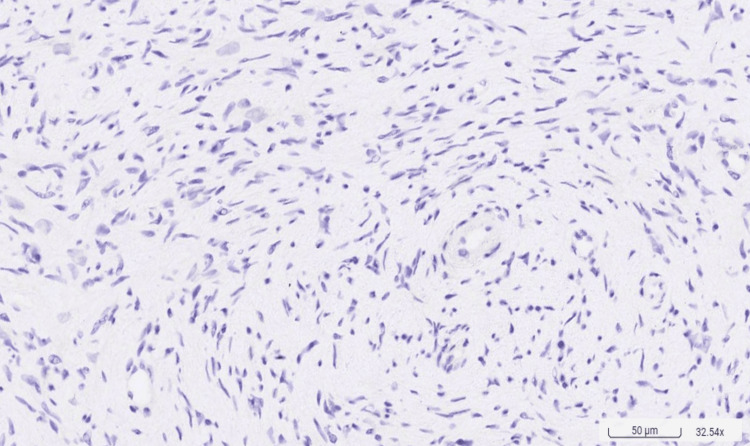
Immunohistochemistry staining indicating negativity for epithelial membrane antigen (EMA) (original magnification x32.54) Negativity for EMA helps exclude other spindle cell neoplasms of epithelial origin.

Clinical assessments, including periodontal probing and examination of the dentition, were normal. Radiographic examination showed no abnormalities. The differential diagnosis of the palatal tumor included pyogenic granuloma, peripheral giant cell granuloma, peripheral ossifying fibroma, and neurofibroma. A biopsy conducted on March 28, 2023, was performed. The histopathological examination revealed the tumor's spindle cellular composition without a definitive cellular arrangement pattern. Immunohistochemical staining is pivotal in diagnosis, with solitary neurofibromas typically exhibiting focal positivity for S100 protein, indicative of Schwann cell involvement, and negativity for epithelial membrane antigen (EMA), which helps exclude other spindle cell neoplasms. The absence of a specific cellular arrangement pattern in histopathology, coupled with the lack of clinical signs of NF1, supports the diagnosis of a solitary neurofibroma. A focally positive S100 stain, in this context, may suggest the presence of a solitary neurofibroma (Figures 2-4).

**Figure 5:**
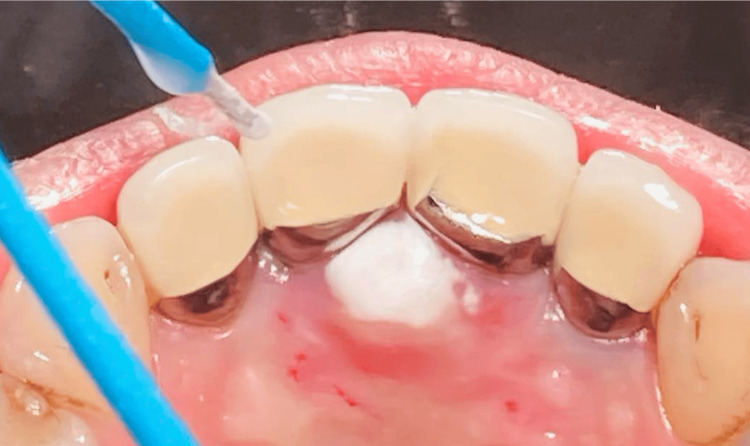
Open-system liquid nitrogen cryotherapy was in action During the cryotherapy session, we utilized the Cry-Ac®-3 B-800 Brymill Cryogun, fitted with a 1-inch 16-gauge straight spray needle. The needle tip was accurately positioned 1 cm away from the lesion at a 90° angle. Liquid nitrogen application was meticulously controlled using the timed spot freeze method to ensure precision. Additionally, a high-power suction device was employed simultaneously to disperse the vapor fog, ensuring clear visibility throughout the procedure. The ice field is maintained at the desired distance (2 mm from the visible tumor margin) throughout the freezing period and naturally recedes during the thawing phase (as indicated by the white area over incisive papilla).

**Figure 6:**
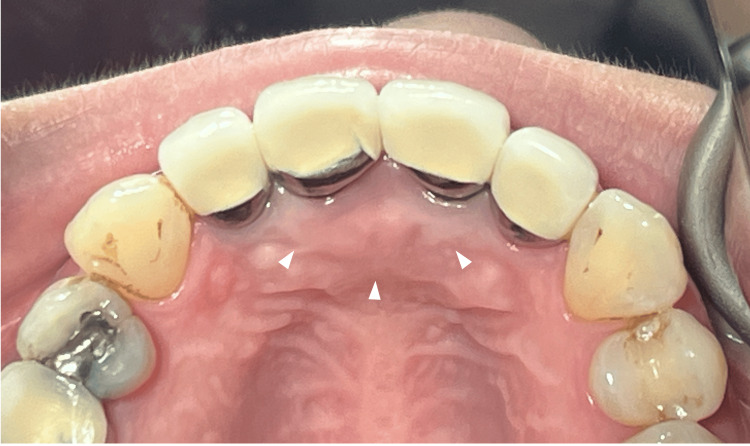
Complete healing of the incisive papilla solitary neurofibroma (white arrow heads) Complete healing, with no recurrence observed during the five-month follow-up.

**Figure 7:**
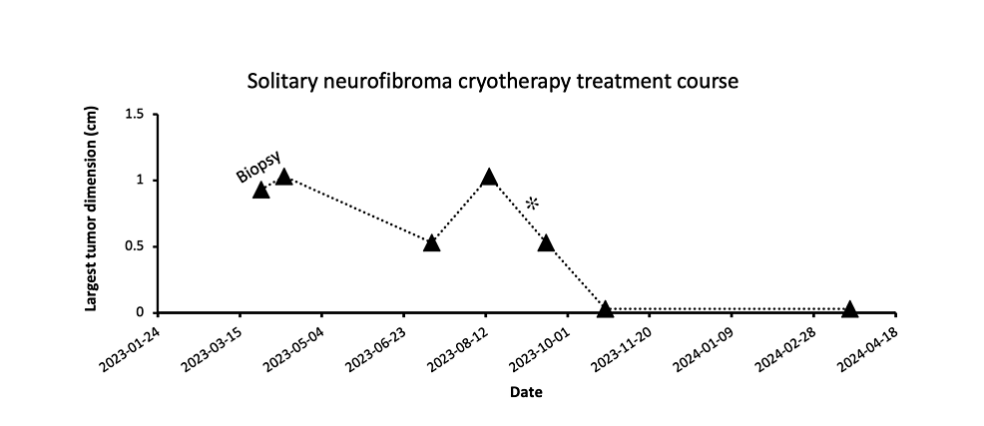
The treatment course of the palatal solitary neurofibroma The clinical treatment course, correlating with tumor size, is depicted in the chart. Following the biopsy, liquid nitrogen cryotherapy sessions consisted of two freeze-thaw cycles of 1-2 minutes, except as indicated by an asterisk, which denotes two freeze-thaw cycles of 2-2 minutes.

No signs of diffuse NF1 were found thereafter. Despite its benign nature, the patient declined surgery and chose LNC. In the cryotherapy session, the Cry-Ac®-3 B-800 Brymill Cryogun, fitted with a 1-inch 16-gauge straight spray needle, was utilized. The needle tip was precisely positioned 1 cm from the lesion at a 90° angle. Liquid nitrogen application was carefully controlled through the timed spot freeze method to ensure precision. A high-power suction device was simultaneously used to dissipate the vapor fog, maintaining clear visibility throughout the procedure. Subsequent LNC treatments on April 11 and July 10, each comprising two 1-2 minute freeze-thaw cycles, resulted in a 10 x 7 mm lesion by August 14, 2023. The third LNC session, with 2-2 minute cycles, addressed refractoriness, reducing the lesion to 5 x 3 mm after one month. Following the fourth LNC session, the tumor was completely eliminated at the one-month follow-up, with no recurrence observed during the five-month follow-up by clinical diagnosis (Figures 5-7).

## Discussion

The case report highlights the successful treatment of a palatal SNF in a 45-year-old woman using LNC. This non-invasive method effectively eliminated the tumor without recurrence at a five-month follow-up, demonstrating LNC as a feasible and patient-preferred alternative to surgery for this specific tumor.

The general mechanism of cryotherapy for tumors involves using liquid nitrogen to induce ultra-low temperatures, rapidly freezing tissues. This freezing process causes cellular damage and local destruction of target lesions. Additionally, cryotherapy dehydrates the lesions and induces vascular injuries, including platelet aggregation, increased blood viscosity, vasoconstriction, microthrombi formation, and tissue ischemia. These combined effects contribute to the effective destruction of neoplasms [4,9].

In our case of palatal SNF, we administered precision cryotherapy using a liquid nitrogen cryogun in an open system. This innovative approach for treating oral SNFs was guided by histopathological findings, particularly indicated when patients are reluctant to undergo surgical excision. The timed spot freeze method was crucial for managing the freezing scope and duration, effectively targeting the persistent tumor [9]. Initially, we adopted the protocol for oral hemangioma treatment, adjusting the freeze-thaw cycle duration based on the tumor's response to address its refractoriness [11]. The treatment was intensified by extending the freezing time from one to two minutes, which increased intracellular ice formation and, consequently, lesion destruction. We also expanded the ice field by 2 mm beyond the tumor margins over two cycles. This deliberate increase in the ice field by 1 mm past the visible tumor boundary incrementally enhanced the therapeutic effect on the deeper tumor areas. Thus, a 2 mm increase in the ice field diameter equated to a similar enhancement in the depth of the freezing impact, highlighting the effectiveness of cryotherapy in managing palatal SNF.

When diagnosing an SNF in the oral cavity, it is imperative to assess for NF1 associations. The presence of NF1 typically suggests a plexiform neurofibroma, characterized by its origin from multiple neurons and a more invasive nature, often leading to a poorer prognosis. Conversely, oral SNFs not linked to NF1 usually represent sporadic cases with a more favorable prognosis. While up to 50% of NF1 patients may develop a plexiform neurofibroma, oral manifestations occur in about 72% of these patients. Thus, differentiating sporadic SNF from NF1-associated neurofibromas is critical for proper management and prognosis. In the case presented, no signs of NF1 were observed, indicating a sporadic form of SNF with a generally better outlook [12].

The patient's satisfaction post-LNC treatment underscores the value of respecting patient autonomy and exploring non-surgical options. While the positive outcome warrants cautious optimism, ongoing monitoring for recurrence is essential due to limited prognostic data. Documenting such rare cases is crucial, as they enrich the evidence base for alternative treatments. Although promising, this single case highlights the need for further research to validate LNC's broader applicability for SNF.

## Conclusions

Treating a palatal SNF with LNC demonstrates a compelling non-surgical alternative that aligns with the patient's preference and shows immense promise. The treatment's tailored flexibility, coupled with the absence of recurrence at the five-month follow-up, underscores its potential. Ensuring an accurate diagnosis between sporadic SNF and NF1-related neurofibromas is vital for appropriate treatment and prognosis. This case contributes valuable data to the scarce evidence on cryotherapy for oral lesions, emphasizing the necessity for additional research to formulate comprehensive treatment protocols.
